# Current Characteristics of REBCO Tapes in 6-Slot TSTC-CICC Under Bending Conditions

**DOI:** 10.3390/ma18020350

**Published:** 2025-01-14

**Authors:** Li Li, Mingzhen Yang, Songzhen Yuan, Shaotao Dai, Tao Ma

**Affiliations:** 1 School of Electrical Engineering, Beijing Jiaotong University, Beijing 100044, China; 23115182@bjtu.edu.cn (L.L.); stdai@bjtu.edu.cn (S.D.); taom@bjtu.edu.cn (T.M.); 2 Electric Power Research Institute of Guangdong Power Grid Corporation, Guangzhou 510180, China; ymz19950818@163.com; 3 Joint Laboratory on Power Superconducting Technology of China Southern Power Grid Co., Ltd., Guangzhou 510623, China

**Keywords:** TSTC-CICC, critical current, bend performance

## Abstract

Embedding stacked HTS tapes into twisted slots is one design approach for constructing fusion conductors. This paper adopts a Cable-in-Conduit Conductor (CICC) structure, utilizing commercially REBCO coated conductors. The cable framework is made of copper and features six helically twisted slots filled with 2G HTS tapes. Two 1 m long samples with twist pitches of 200 mm and 300 mm, respectively, were fabricated. In one slot, copper and superconducting tapes were alternated, while the remaining grooves were filled with copper tapes. The 90 µm thick copper-plated bare tapes provided by Shanghai Superconductor were used for testing. By measuring the critical current of tapes positioned at different locations within the grooves at 77 K, the characteristics of each tape in the stacked arrangement were individually characterized. The study obtained the current degradation patterns of tapes located at different positions within the grooves under various bending radii. This paper will present and discuss the preliminary results of the bending measurements conducted at 77 K under a self-field.

## 1. Introduction

The second-generation high-temperature superconductor (2G HTS) rare-earth barium copper oxide (REBCO) has demonstrated immense potential in the field of fusion energy due to its exceptional current-carrying capacity and mechanical stability [1,2]. With continuous advancements in materials science, the application prospects of REBCO are becoming increasingly broad, especially in the design of fusion reactor magnet systems, where its feasibility is becoming apparent [3]. Fusion research projects worldwide, such as EUROfusion DEMO [4], China Fusion Engineering Test Reactor (CFETR) [5], and the Affordable, Robust, Compact (ARC) advanced tokamak [6], are actively exploring the integration of HTS tapes into their technological roadmaps, aiming to leverage this material to advance fusion energy technology. Due to the flat, tape-like structure of 2G HTS tapes, conventional cable technology’s round conductors are challenging to utilize effectively. Compared to low-temperature superconductors like NbTi and Nb3Sn, the tape structure exhibits strong anisotropy, with different mechanical and electromagnetic properties in different directions, posing new challenges for conductor design and fabrication.

Over the past decade, various institutions have proposed and are developing several viable HTS tape wiring techniques [7], including Conductor on Round Core (CORC) cables [8], Roebel Assembled Coated Conductors (RACCs) [9] and various stacked-tape cables including the Twisted Stacked-Tape Cable (TSTC) developed at the Massachusetts Institute of Technology (MIT) [10,11], the Cross-Conductor cable (CroCo) concept proposed by the Karlsruhe Institute of Technology (KIT) [12], and the quasi-isotropic strand [13]. Based on existing structures, MIT and the Italian National Agency for New Technologies, Energy and Sustainable Economic Development (ENEA) have proposed a TSTC-based CICC structure by embedding stacked HTS tapes into a framework [14,15]. This approach not only increases the current-carrying capacity of the conductor but also reduces its anisotropy through optimization of the tape arrangement. The application of vacuum pressure impregnated, insulated, partially transposed, extruded, and roll-formed (VIPER) cable in the SPARC project has demonstrated that this conductor format can meet the fundamental requirements in fusion magnets [16].

This paper designs a six-slot conductor based on the TSTC-CICC structure, inspired by the VIPER design, with an optimized number of slots. This design aims to achieve higher current-carrying capacity by increasing the number of 2G HTS tapes. A higher current-carrying capacity can reduce the volume of fusion magnets, thereby significantly lowering the overall construction cost of fusion systems. However, as the number of slots increases, the resulting larger conductor diameter may affect its bending performance, which contradicts the design goals of compact fusion magnets. During the process of winding the conductor into a coil, it is inevitable to bend the conductor. Since the REBCO material in the superconducting tape belongs to a ceramic thin film, the deformation generated during the bending process will lead to irreversible cracks in the material and ultimately affect its electrical conductivity [17]. Especially for the CS and TF magnets in compact fusion systems, where the minimum bending radius is only a few tens of centimeters, it is crucial to deeply study the performance of the conductor under bending conditions. To evaluate the conductor’s bending performance, this study fabricated samples with two different twist pitches and measured the critical current of tapes at various positions within the slots under different bending radii. Through these experiments, we obtained the variation patterns of the current in tapes during bending. The experimental results indicate that the bending radius of the six-slot TSTC-CICC meets the basic requirements for fusion magnets. Additionally, the manufacturing process of the conductor can be designed using existing technological pathways, providing a basis for the industrial-scale production of long conductors.

## 2. Conductor Fabrication and Experimental Setup

**Figure 1:**
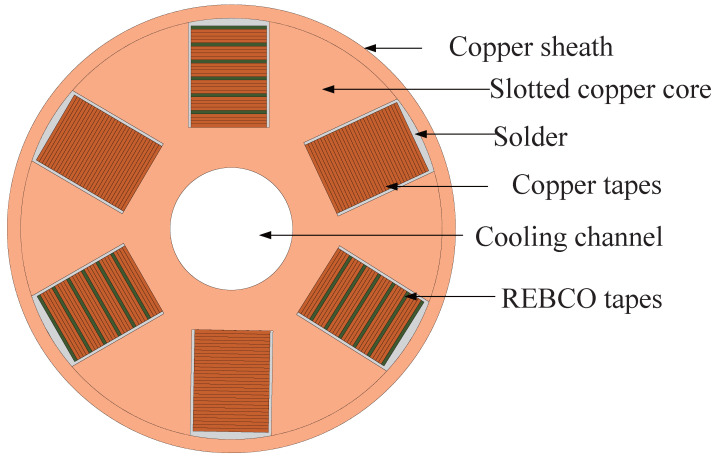
The figure shows the cross-section of a six-slot TSTC-CICC, where HTS tapes are located in three symmetrically positioned slots, and copper tapes are used to evenly space the tapes. The remaining slots are filled with copper tapes of the same thickness, and the outermost part is fixed by the copper sheath.

Based on the existing conductor design, this paper proposes a six-slot TSTC-CICC layout, as shown in Figure 1. It is expected that under its self-field, when all slots are filled with HTS tapes, the critical current can reach approximately 18 kA at 77 K, or it can achieve a current-carrying capacity of over 60 kA under conditions of 4.2 K and 12 T [18]. The cable consists of a central copper framework and stacked HTS tapes with six helical slots on the framework for embedding the tapes. The framework has an outer diameter of 25 mm, including a central channel with a diameter of 6 mm, specifically designed for coolant flow to maintain thermal stability under current flow conditions. The slots have a rectangular cross-section of 4.5 × 7 mm, which is suitable for standard 4 mm wide HTS tapes. It is particularly noted that due to differences in the twist pitch, the slot width on the conductor’s cross-section varies with the twist angle. The slot width mentioned in this text refers to the width in the direction perpendicular to the helix.

The number of HTS tapes that can be accommodated in the slots varies depending on the thickness of the tapes: if the tape thickness is 0.1 mm, 60 tapes can be embedded; if the thickness increases to 0.15 mm, 40 tapes can be embedded. After each tape is embedded into the groove, it is tightly wrapped with a 50-micron copper foil to prevent the tape from spilling out of the slot, ensuring the integrity of the structure. The outermost sheath provides the necessary mechanical support, ensuring the stability of the conductor under various working conditions. During the solder impregnation process, while the mechanical strength of the conductor is enhanced, its bending performance may decrease. Therefore, an effective strategy is to bend the conductor into the desired shape first, which usually takes place during the magnet winding process [19]. Then, solder is used to fill the gaps in the slots, ensuring a defect-free conductor structure, which maintains the necessary bending performance while ensuring mechanical strength.

**Figure 2:**
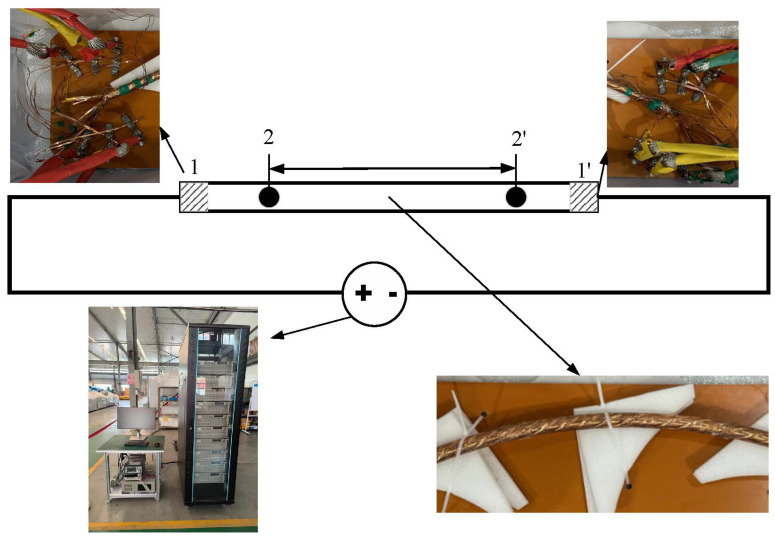
The figure shows a schematic diagram for critical current testing. In the diagram, 1-1′ indicates the position where the current is introduced into the superconducting tape, 2-2′ represents the position where the voltage on the tape is measured, and the rectangular frame is the conductor after bending.

Under the conditions of 77 K (liquid nitrogen) and self-field, we measured the I–V curve of the bent superconducting tapes, using 1 μV/cm as the criterion for critical current. As shown in Figure 2, the current contact was achieved by soldering copper sheets to the ends of the tapes and pressing them firmly between two copper plates. The voltage probes were placed at the ends of the tapes, spaced 100 cm apart, to ensure measurement accuracy. Since the contact resistance between the tapes is much higher than the solder resistance, and the resistance of copper is also significantly higher than that of the REBCO layer, the current does not transfer to other layers through the contact resistance before the superconducting tape quenches. This ensures that the current primarily flows through the HTS tapes until it exceeds the critical state. During the test, we injected current into the HTS tapes and recorded the corresponding voltage drop. Through this method, we were able to accurately measure the critical current of each tape, thereby assessing its performance in the bent state.

## 3. Analysis Modeling

**Figure 3:**
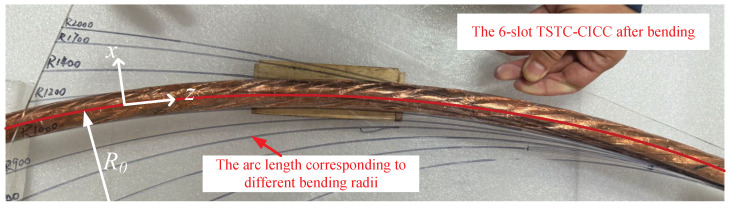
A three-point bending process was conducted using a pipe bender, gradually reducing the bending radius of the conductor, and the bending radius was determined by measuring the arc length of the bent conductor. After determining the bending radius, the I–V curves of the tapes at different positions were measured.

In the case shown in Figure 3, when the six-slot TSTC-CICC undergoes bending, the cable’s bending neutral axis is positioned exactly at the center of its axis (represented by the red solid line) and remains perpendicular to the bending plane. Consequently, the tape on the outside of the cable’s neutral plane experiences tensile force, while the inner portion is subjected to compressive force. To reduce the conductor’s bending radius, the tapes are stacked and embedded within a helical groove, causing the strain experienced by tapes in different positions to exhibit periodic variation during bending. It should be noted that the cable’s neutral plane is a circular plane defined by the cable’s neutral axis, which is perpendicular to the cable’s bending plane. Since the thickness of the superconducting layer within the tape is significantly smaller than other dimensions (such as tape width, cable diameter, and cable bending radius), the effect of tape thickness on bending strain can be neglected in calculations.

**Figure 4:**
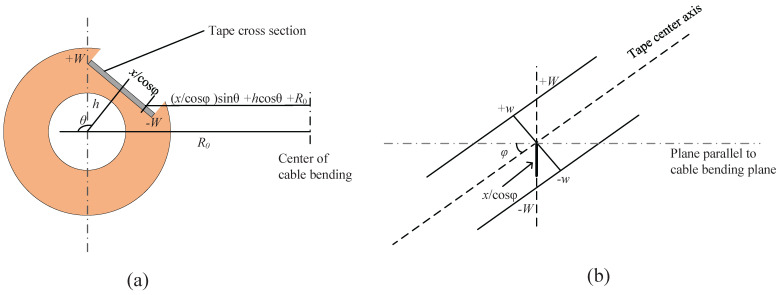
Model schematic of the REBCO layer strain during bending in a six-slot TSTC: (**a**) cross-sectional view of the tape embedded in the groove, using the bottom tape as an example; (**b**) enlarged view of a segment of the tape after twisting, embedded in the helical groove framework.

In Figure 4a, the distance between the center of the tape and the center axis of the cable is defined as h (tape off-center distance), which varies for tapes at different positions within the groove. Figure 4b further shows an enlarged view of a segment of the tape embedded in the groove and twisted, representing an example of stacked tape within the groove. The actual width of the tape is denoted as *w*, while the width of the tape cross-section after twisting is represented as *W*. The position change of the tape in the width direction is represented by *x*. As shown in the figure, the calculation formula for axial strain along the width direction of the tape at different bending radii is provided.

(1) $$\varepsilon_{b}=\frac{xsin\theta +hcos\theta cos\varphi }{R_{0}}$$

In the formula, θ represents the twist angle, and φ is the angle of twist caused by the helical groove in the framework, which is determined by the twist pitch and the distance *h* from the center to the tape. The specific calculation process is shown in the following formula.

(2) $$\varphi =\tan^{-1}\left(2\pi h/L_{p}\right)$$

(3) $$\theta =\frac{2\pi h\cos\varphi }{L_{p}}$$

Notably, before conducting bending tests, the actual tape experiences a certain torsional load during embedding into the framework. However, according to the calculation formula provided in this paper, the strain caused by twisting is negligible compared to that induced by bending. Therefore, this study mainly focuses on the additional strain imposed on the tape by the bending process. Specifically, when the twist pitch exceeds 200 mm, the impact of torsional strain on the critical current of the 4 mm thick REBCO tape TSTC conductor is much less significant than that of bending strain.

During the conductor manufacturing process, the relative slippage allowed between tapes enables us to simplify Equation (1). When stacked tapes can freely slide within the groove during bending, they can release longitudinal strain through this slippage. Inside the conductor, due to the twisting characteristics of the tape, compressive and tensile stresses achieve equilibrium across the twist pitch and cancel each other over its short length, which is a phenomenon that applies similarly to long cables. Therefore, the longitudinal strain caused by the second term in Equation (1) can be reduced by multiplying by a coefficient *k* (related to the friction coefficient between the tapes). In this scenario, the bending strain $\varepsilon_{b}$ calculated from Equation (1) is correspondingly reduced, as shown below:

(4) $$\varepsilon_{b}=\frac{xsin\theta +khcos\theta cos\varphi }{R_{0}}$$

It is evident from the equation that when considering relative sliding between the tapes to release strain, the strain in the tapes is reduced compared to the original formula.

## 4. Results and Discussion

**Figure 5:**
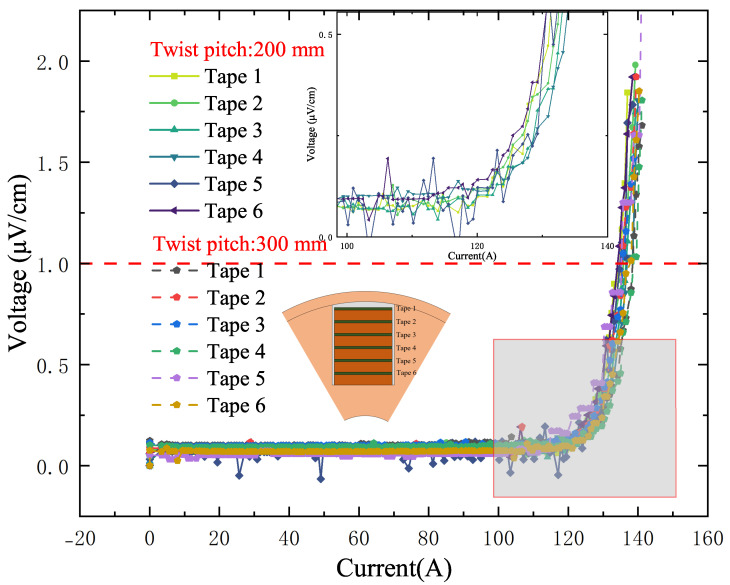
The I–V curves of the tapes at different positions in the groove when the tapes are embedded in the skeleton but in an unbended state.

Figure 5 shows the *I–V* curve measurement results for each individual HTS tape during the stacking process of samples with two different twist pitches. The six superconducting tapes in the slot are evenly distributed, with a 1 mm gap between them filled with copper tapes, and the critical current of each tape in the straight cable state was measured. It is evident from the figure that the measured critical current values align with the specifications provided by the tape supplier with the measured critical current ranging between 130 and 140 A. This result indicates that the strain applied to the superconducting tapes by the 200 mm and 300 mm twist pitches did not reduce their current-carrying capacity. The observed reduction in critical current during the experiment is directly attributed to the bending of the conductor rather than the twisting.

**Figure 6:**
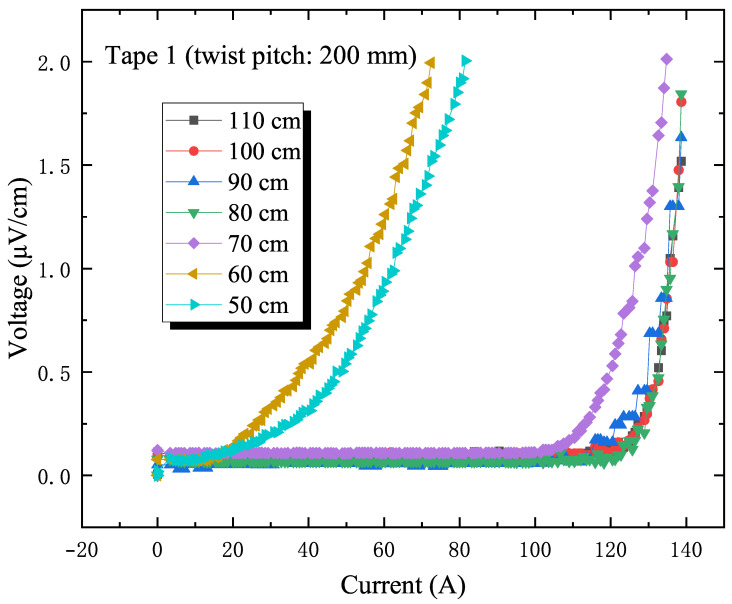
I–V curves of tapes within the slot of the 200 mm twist pitch sample under different bending radii.

**Figure 7:**
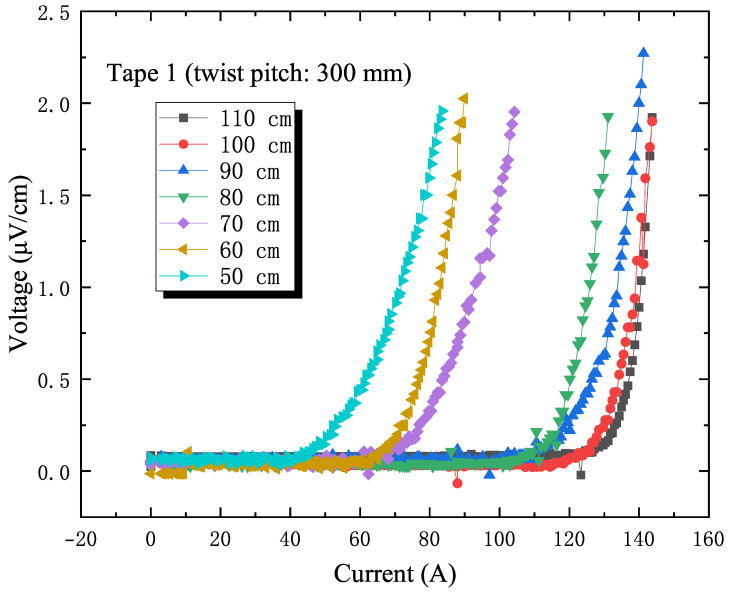
I–V curves of tapes within the slot of the 300 mm twist pitch sample under different bending radii.

The conductor bending experiment revealed a phenomenon: in the bent state, the current degradation experienced by the stacked superconducting tapes in the slot is unevenly distributed. According to the bending strain calculation formula ε=y/R (y is the distance from the axis, R is the bending radius), we can infer that the farther a tape is from the neutral axis, the greater the strain induced by bending. As shown in Figure 6 and Figure 7, this theoretical prediction is consistent with experimental observations [20]: among the stacked tapes in the slot, the outermost tape (labeled as Tape 1) was the first to show current degradation. Therefore, the current degradation of the outermost tape under bending is a key focus of study.

Figure 6 and Figure 7 detail the I–V curves of tape 1 in the slot. For the conductor with a 200 mm twist pitch, tape 1 began to show current degradation at a bending radius of 70 cm. When the bending radius was further reduced to 60 cm, the magnitude of the current drop increased significantly, far exceeding the initial drop. For the sample with a 300 mm twist pitch, the increased pitch lengthened the transposition length of the stacked tapes in the conductor, which somewhat affected its adaptability to bending. The experimental results also confirmed this: the sample with a 300 mm twist pitch showed a slight current drop at a bending radius of 90 cm with a sharp drop at 70 cm. The I–V test curves are usually fitted by using the E–J characteristic curves:

(5) $$E=E_{0}\frac{J}{J_{c}\left(B\right)}{\left|\frac{J}{J_{c}\left(B\right)}\right|}^{n-1}$$

where $E_{0}$ represents the critical electric field (1 μV/cm), and $J_{c}\left(B\right)$ represents the relationship between the critical current density and the magnetic field. The *n*-value is a fitting parameter based on the voltage curve of the superconducting material, which is typically ranging in the tens. A larger *n*-value means that the superconducting tape is more rapid during the quench transition process. Additionally, after the current degradation in both samples, their *n*-value decreased to varying degrees. Before the conductor was bent, the n-value of the tapes in the slot was estimated to be between 30 and 40. However, once the current began to degrade, the n-value dropped to between 20 and 30.

**Figure 8:**
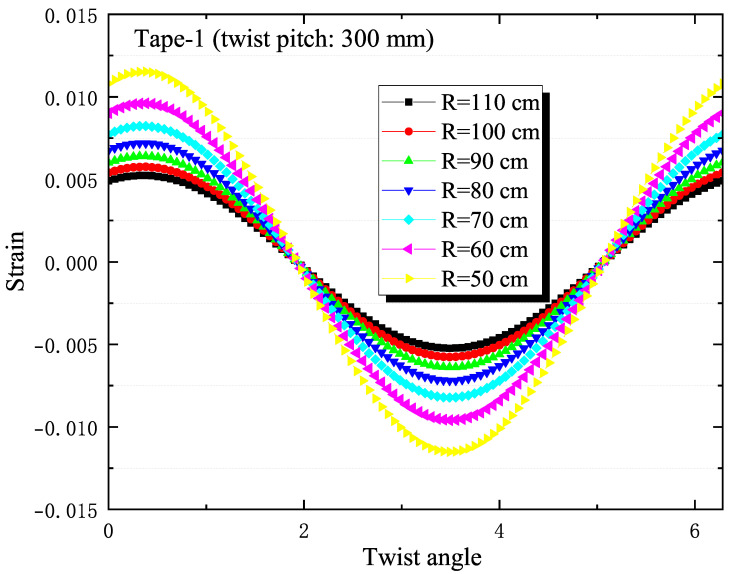
The figure shows the analytical calculation results of the axial strain of the outermost tape in the groove under different bending radii.

**Figure 9:**
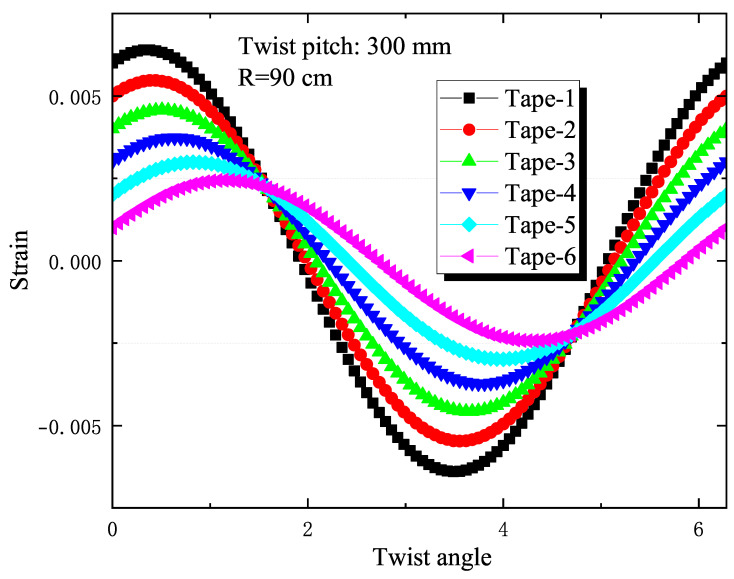
The figure shows the axial strain calculation results of the tape at different positions in the groove for a sample with a twist pitch of 300 mm at a bending radius of 90 cm.

In Figure 8, the analytical calculations display the strain of the outermost tape (tape 1) in the slot under various bending radii. The bending strain is assessed using Equation (4), which correlates the twist angle θ with different bending radius values and compares it to experimental results. From the calculations, it is observed that as the bending radius decreases, the calculated strain values of the outermost tape show a periodic reduction trend, which aligns with the experimental findings. Figure 9 presents the axial strain values of tapes at different positions within the slot for a sample with a twist pitch of 300 mm and a bending radius of 90 cm. The results indicate that the closer the tape is to the neutral axis, the lower its calculated strain value, which is also consistent with the experimental results.

The strain distribution of the tapes within the slot during the bending process exhibits complexity, which is a topic that requires in-depth research. Since the solder curing process is performed after the conductor is bent, the stress release caused by sliding between the tapes during bending occurs naturally, which is beneficial for the conductor to bend smoothly. To thoroughly understand the proposed design and to comprehensively evaluate the cable’s performance, it is necessary to conduct more in-depth experimental exploration and numerical simulations using an FEA model.

## 5. Conclusions

This paper investigates the current characteristics of a six-slot TSTC-CICC based on 2G HTS tapes under bending conditions. By designing and fabricating two 1 m long samples with different twist pitches (200 mm and 300 mm), we measured the changes in the critical current of superconducting tapes at different positions within the conductor slot under various bending radii. The experimental results indicate that the current degradation of superconducting tapes under bending conditions is unevenly distributed. Specifically, the superconducting tape farthest from the neutral axis is the first to show current degradation. Additionally, we found that after current degradation begins, the n-value of the superconducting tapes decreases, dropping from 30–40 before bending to between 20 and 30. This phenomenon may be related to the complex strain distribution within the superconducting tapes during bending, requiring further research for a deeper understanding. In conclusion, the six-slot TSTC-CICC meets the basic design requirements for fusion magnets. Future work will involve more in-depth experimental exploration and numerical simulations to comprehensively evaluate and optimize the performance of this conductor.

## Data Availability

The original contributions presented in this study are included in the article. Further inquiries can be directed to the corresponding author.
